# Common Misconceptions about PCBs Obscure the Crisis of Children’s Exposure in School

**DOI:** 10.1021/acs.est.2c07943

**Published:** 2022-11-18

**Authors:** Keri C. Hornbuckle

**Affiliations:** Department of Civil and Environmental Engineering and IIHR-Hydroscience and Engineering, University of Iowa, Iowa City, Iowa52242, United States

**Keywords:** PCB congeners, emissions, schools, remediation, building materials, exposures

## Introduction

**Figure 1:**
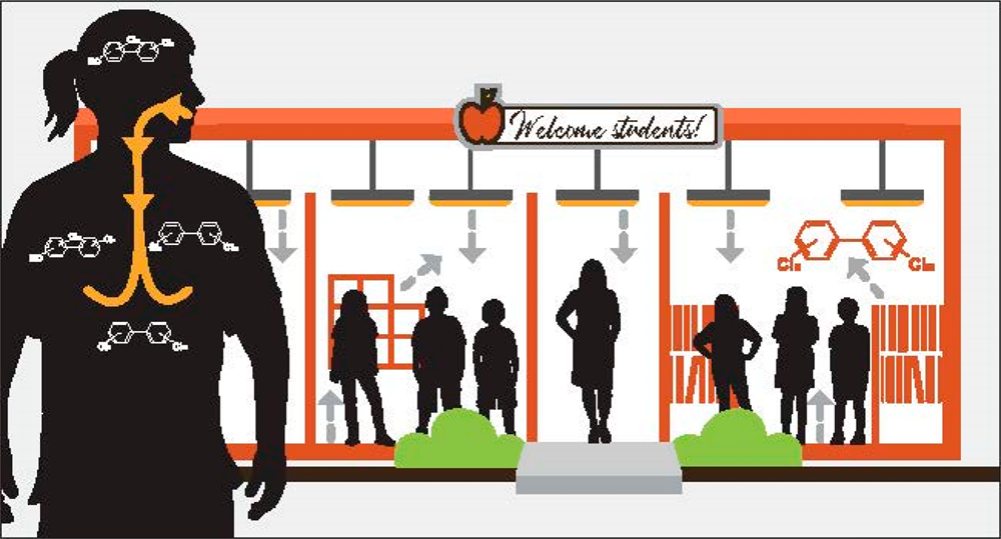
I identify eight commonly held misconceptions about polychlorinated biphenyls (PCBs). These misconceptions contribute to ongoing human exposures to airborne PCBs in schools.

Polychlorinated biphenyls make up a set of 209 environmental contaminants that have been subject to a great deal of attention for more than 50 years. They are perhaps the most famous of the Stockholm Convention’s “Dirty Dozen” and the most widely recognized of the chemicals called “Legacy” or “Forever Chemicals”. I have studied PCBs in the environment for more than 30 years, and recently, my collaborators and I have shown that PCBs in school air pose a clear health hazard to children and people who work in schools (Figure 1). We have found that many schools harbor surprisingly high levels of these toxic compounds. We are not alone in noticing the high concentrations of PCBs in schools. Families, teachers, attorneys, and politicians are demanding attention. For example, in October 2022 a jury awarded $275M to 13 children and their families who attended a PCB-contaminated school. In February 2022, Senator Ed Markey and others sent a formal letter to urge the U.S. Environmental Protection Agency to support funding to address PCBs in schools. However, a high level of misunderstanding about PCBs persists, even among scientists. These misconceptions are not benign but in fact result in long-term harm to our population. These misconceptions support inaction to protect the public and contribute to an ongoing and highly unjust assault on health. In this article, I identify and debunk eight commonly held opinions about PCBs.

### Misconception 1. Living near a PCB Superfund Site Is the Worst Case for Human Exposure

*Our research has shown that the worst case for human exposure is a school room*. We have measured airborne PCBs near some of the largest PCB-contaminated sites in the United States, including over the contaminated waters of Green Bay and the Indiana Harbor and Ship Canal in Lake Michigan, and at New Bedford Harbor in Massachusetts. We have also measured airborne PCBs throughout the Chicago metro area. The highest level we recorded was 38 ng/m^3^, immediately adjacent to New Bedford Harbor, one of the largest PCB Superfund sites in the country, yet concentrations we measure in schools have exceeded this value. Our studies of schools indicate that concentrations are equal or higher in schools built or remodeled during the PCB era.^1−3^ Because of the numbers of children affected, I now believe attending or working in these schools represents the worst case for human exposure.

### Misconception 2. The Use of PCBs Is Banned

In the United States, PCBs were not banned from use. PCBs, including Monsanto’s Aroclor mixtures, remain in the materials for which they were originally designed. They are still found in the transformers that hang from poles in our yards, and they still reside in buildings constructed during the PCB era between 1950 and 1980. One of the easiest of PCB building materials to remove is fluorescent light ballasts, but Aroclor PCBs were also added to the adhesives under floor tile, to masonry sealants, to window caulking, and to the glaze used between glass blocks frequently found in school rooms. These sources of PCBs are more difficult to identify and remove and were used in school building materials.

### Misconception 3. PCBs in Air are Declining

Because Aroclor PCBs are present in so many surface materials and because “closed” systems like light ballasts are not truly airtight, and because so many of these materials were used indoors, PCB concentrations in buildings built during the PCB era have increased levels of airborne PCBs. Until they are removed, Aroclor mixtures used in school building materials will continue to degas PCBs into the room air.

### Misconception 4. Diet, and Especially Fish, Is the Only Important Route of Exposure

My colleagues and I have measured PCBs in the air inside and outside homes and schools, and in food. We found exposure through inhalation was similar in magnitude to that from diet.^2−4^ This surprising result is partly explained by the low level of PCBs in processed foods and low level of consumption of fish in our study participants. For many children, their exposure to PCBs was primarily due to high levels of gas-phase PCBs in school air.

### Misconception 5. Only Dioxin-like PCBs Are Toxic

PCBs are now categorized as known human carcinogens, and dioxin-like congeners have the highest potency. Unfortunately, non-dioxin-like PCBs have also been associated with neurological impairments, including autism and attention deficit and anxiety disorders. Children and adolescents are particularly vulnerable, and the PCBs associated with these effects are found in school air.

### Misconception 6. PCBs Do Not Break Down

Many PCB congeners are readily transformed through biological processes to other compounds, called PCB metabolites. As a result, the mixture of PCBs to which we are exposed does not resemble the mixture found in our blood. Notably, the lower-molecular weight congeners commonly found in school air are rapidly metabolized to OH-PCBs, PCB sulfates, PCB methyl sulfones, PCB glucuronides, and other compounds. Unfortunately, these PCB metabolites have toxicities similar to those of PCBs.

### Misconception 7. Lower-Molecular Weight PCBs Are Harmless

Many lower-molecular weight PCBs found in school air rapidly convert to metabolites. Although additional research is needed, studies indicate that the metabolites of less chlorinated PCBs are the more direct cause of toxicity, including neurological effects.

### Misconception 8. The Superfund Legislation Provides a Means to Clean Up PCBs and Reduce Human Exposure

Schools are responsible for their own buildings. Although it is now clear that children are among the most vulnerable to PCB toxicity, and although we know PCBs were heavily used in building materials and we know that more than 50 000 schools were built or remodeled during the PCB era of 1950–1980, there is still no federal funding available for the cleanup of PCBs in schools. The Superfund legislation, known formally as the Comprehensive Environmental Response, Compensation, and Liability Act (CERCLA), provides a means to clean up PCBs in certain environmental sites. Through CERLA and subsequent legislation, billions have been spent or will be spent on cleanup of PCB-contaminated sediments, but PCB remediation of schools is left to the states, municipalities, school districts, and officials of the individual schools. Because most schools are funded at the local level through property and sales taxes, the opportunity for PCB remediation of schools is deeply unfair. Because of this unfair access to remediation funds, only a few schools have been remediated at a per-school cost of millions of dollars. I hope this will change. Surely, there is an opportunity to use what we now know about PCBs to direct funds to this most important need.

To be sure, additional research about PCBs in school air is urgently needed. A better understanding of the toxicity associated with inhalation of all of the PCB congeners found in school air is needed. A better understanding of the toxic effects of PCB metabolites is needed. What is the impact of 12 years of PCB inhalation? But ignorance of complete toxicological effects should not slow progress in reducing children’s exposure to PCBs. We also need research to support more cost-effective methods for physical remediation of PCB-contaminated buildings. Current methods for school building PCB abatement use wipe tests, which are mostly qualitative, and Aroclor analyses. A more refined approach that links the sources of air emissions to the concentrations in air experienced in the school is needed. Congener-specific analysis is often helpful, and direct measurement of emissions can help prioritize and focus remediation on the removal of the largest emission sources.
